# Technical note: Commissioning of a low‐cost system for directly 3D printed flexible bolus

**DOI:** 10.1002/acm2.14206

**Published:** 2023-11-14

**Authors:** Garrett C. Baltz, Steven M. Kirsner

**Affiliations:** ^1^ Scripps Cancer Center San Diego California USA

**Keywords:** 3D printing, bolus, dosimetry, electron radiotherapy, treatment planning

## Abstract

**Purpose:**

To present the commissioning process of a low‐cost solution for directly 3D printed flexible patient specific bolus.

**Methods:**

The 3D printing solution used in this study consisted of a resin stereolithography 3D printer and a flexible curing resin. To test the dimensional accuracy of the 3D printer, rectangular cuboids with varying dimensions were 3D printed and their measured dimensions were compared to the designed dimensions. Percent Depth Dose (PDD) profiles were measured by irradiating film embedded in a 3D printed phantom made of the flexible material. A CT of the phantom was acquired and used to replicate the irradiation setup in the treatment planning system. PDDs were calculated for both the native HU of the phantom, and with the phantom HU overridden to 300 HU to match its physical density. Dosimetric agreement was characterized by comparing calculated to measured depths of R90, R80, and R50. Upon completion of the commissioning process, a bolus was 3D printed for a clinical case study for treatment of the nose.

**Results:**

Dimensional accuracy of the printer and material combination was found to be good, with all measured dimensions of test cuboids within 0.5 mm of designed. PDD measurements demonstrated the best dosimetric agreement when the material was overridden to 300 HU, corresponding to the measured physical density of the material of 1.18 g/cc. Calculated and measured depths of R90, R80, and R50 all agreed within 1 mm. The bolus printed for the clinical case was free from defects, highly conformal, and led to a clinically acceptable plan.

**Conclusion:**

The results of the commissioning measurements performed indicate that the 3D printer and material solution are suitable for clinical use. The 3D printer and material combination can provide a low‐cost solution a clinic can implement in‐house to directly 3D print flexible bolus.

## 1 INTRODUCTION

The use of 3D printers to fabricate patient specific bolus has gained widespread general use in radiation oncology.^1^, ^2^ There are multiple commercial vendors that provide solutions to enable clinics to 3D print bolus in house, or print the bolus for the clinic as a service.^3^, ^4^ The most prevalent 3D‐printing technology used is fused deposition modeling (FDM), where a spool of material is melted and extruded in layers to create the object. FDM printers are best suited for printing with rigid plastic materials such as polylactic acid (PLA) and acrylonitrile butadiene styrene (ABS), or some semi‐flexible thermoplastic polyurethane (TPU) materials like Ninjatek Cheetah.^5^ Fabricating patient‐specific boluses using FDM 3D printers has proven to be a cost‐effective and efficient method. However, the resulting boluses are rigid and non‐elastic, which cause patient discomfort and non‐conformality if the patient's anatomy differs from the reference scan.

Due to the limitations of directly 3D printed rigid boluses, there have been techniques developed to use 3D printing to fabricate more flexible bolus. One such technique is 3D printing a mold to cast a silicone bolus.^6^, ^7^, ^8^, ^9^ Compared to conventional 3D printed boluses, the silicone is softer and has improved flexibility, which is more comfortable to the patient and can offer improved conformality. However, the major limitation of this technique is it is very labor intensive due to the multistep process of 3D printing a mold, mixing the silicone, pouring the silicone into the mold, and finally waiting for the silicone to cure. Another method that has been presented in the literature utilizes a different 3D‐printing technology, PolyJet 3D printers, which have the capability to directly 3D print materials with high flexibility and softness similar to silicone.^10^, ^11^ While this technology reduces the burden of having to cast silicone with 3D printed molds, the printers remain prohibitively expensive for clinics to purchase to have one in‐house. Additionally, ordering a bolus from a 3D‐printing company using this technology could also be cost prohibitive. Based on the limitations of these techniques, there is a need for a cheaper and less labor‐intensive technique to use 3D printers for the fabrication of flexible bolus.

The continued development of 3D printers as a mass consumer technology has reduced the cost of many advanced 3D printing technologies, enabling new techniques for 3D printing boluses. One such technology is stereolithography (SLA) using liquid‐crystal displays (LCDs). LCD SLA 3D printers print objects by using the LCD to display a mask that selectively exposes UV light to cure liquid resin in a vat. Like PolyJet printers, LCD SLA 3D printers are resin‐based and can directly print soft and flexible objects. However, they are significantly less expensive, with small printers available for as little as $300. This technology allows clinics to have the ability to directly 3D print flexible boluses in‐house. In this work, we present an example of an in‐house SLA 3D printer and material for directly 3D printing flexible bolus, and present the commissioning process to implement it clinically.

## 2 MATERIAL AND METHODS

### 2.1 3D printer and material

The 3D printer used in this work is the Anycubic Photon M3 Max (Anycubic, Hong Kong). This printer is an LCD SLA printer with a 13.6″ monochrome 7k LCD screen enabling it to print with an in‐plane resolution of 46 μm and a layer height of 0.01–0.1 mm. The printer has a build volume of 30 × 29.8 × 16.4 cm.

The material used in this work is Liqcreate Premium Flex resin (Liqcreate, Utrecht, The Netherlands). This material has a shore value of 63A, giving it excellent flexibility and softness similar to silicone.

### 2.2 Dimensional accuracy

Characterization of the dimensional accuracy of the printer is critical to ensure the printed bolus is conformal to the treatment area and is representative of how it was planned in the treatment planning system (TPS). To assess the dimensional accuracy, test rectangular cuboids with known dimensions were designed in Tinkercad software (Autodesk Inc., San Francisco, CA) and exported as .stl files. A total of four test cuboids were designed and printed with the following dimensions (height, length, width) in mm: 20 × 20 × 20, 5 × 40 × 20, 20 × 80 × 60, 80 × 20 × 60.

There are many open‐source and freely available slicing software to create print files from .stl files for LCD SLA 3D printers. However, it has been reported that the dimensional accuracy of the printed part can vary depending on the slicing software, material, and 3D printer combination used.^12^ Therefore, it is imperative that whatever combination used is characterized. The slicing software used in this work was Anycubic Photon Workshop v3.1.0, which is developed by the same company that manufactures the 3D printer and has pre‐configured profiles specific for the M3 Max printer.

While the Photon Workshop software has pre‐configured profiles of the printing parameters for many commonly used resin materials, the Liqcreate Premium Flex material required a manually tuned profile in order to successfully print reliably. The manufacturer of the material provides suggested printing parameters on their website;^13^ however, these had to be further tuned after a series of test prints and failures. The final printing parameters used to print the cuboids and all other parts in the current study are presented in Table 1.

**TABLE 1:** Final optimized printing parameters used in the present study.

Parameter	Value
Layer thickness	0.1 mm
Exposure time	6 s
Off time	2 s
Bottom exposure time	75 s
Number of bottom layers	3
Lift height	8 mm
Lift speed	1 mm/s
Retract speed	1.5 mm/s
Support type	Heavy
Support angle	50°
Resin temperature	25°C

3D printed resin parts require post processing steps of washing and curing before the part can be used. The washing step removes any leftover liquid resin on the part and was performed by washing the part in a bath of 99% isopropyl alcohol for 6 min using the Anycubic Wash and Cure Plus. After washing, the part needs to be cured with a UV lamp to completely cure the resin throughout the part. The parts were cured for 30 min, also using the Wash and Cure Plus.

Following post processing, the dimensions of the printed cuboids were measured using an iGaging EZ Cal Digital Caliper (San Clemente, CA) with a calibrated uncertainty of 1.5 μm. The mass of the printed cuboids was measured and used to calculate the density.

### 2.3 Dosimetric characterization

Published literature indicates that 3D printing materials do not typically fall within the standard clinical Hounsfield unit (HU)‐to‐Electron density curves.^5^ Therefore, these materials must be dosimetrically characterized.

**FIGURE 1:**
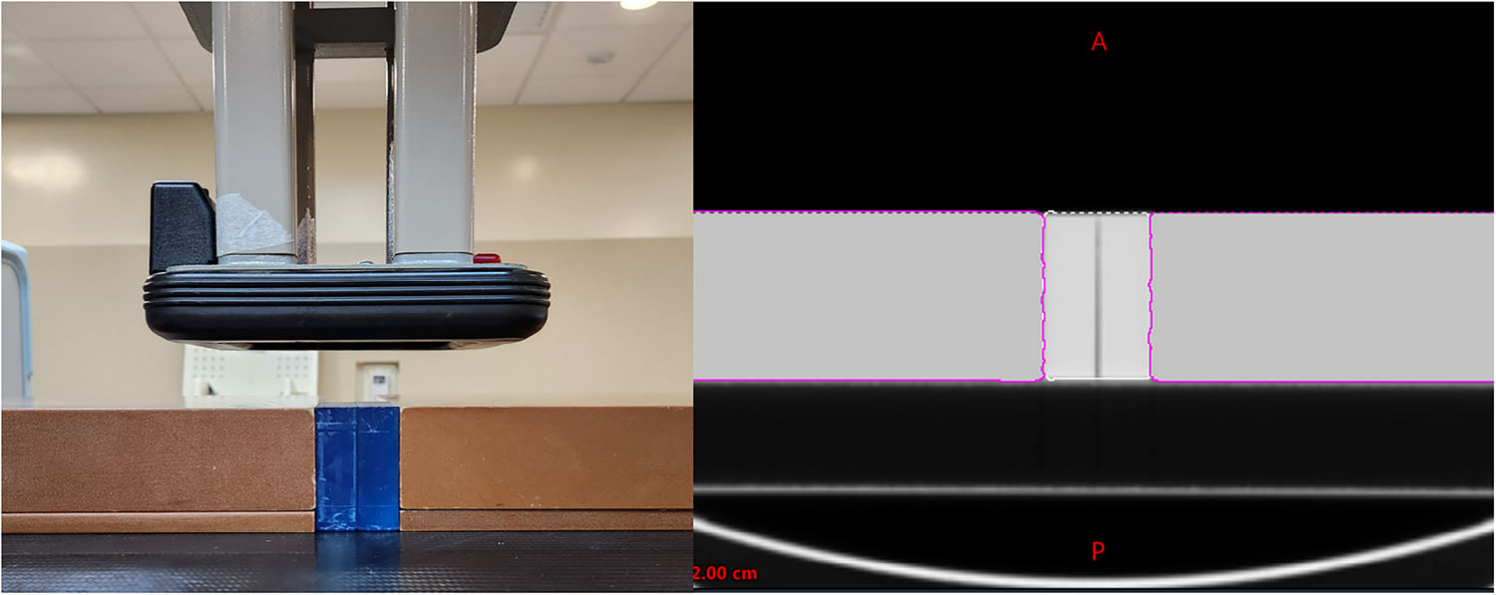
Photos of the phantom setup used for PDD film irradiations. Left image shows the physical setup used for irradiating the films. Right image shows a CT scan of the phantom in the TPS.

Percent Depth Dose (PDD) curves were measured using film in a phantom of the Premium Flex material, presented in Figure 1. The phantom was fabricated by 3D printing two solid blocks with dimensions of 3 × 8 × 6 cm. Gafchromic EBT3 film (Ashland, Bridgewater, NJ) cut into a 2.5 × 6 cm strip was sandwiched between the two blocks aligned parallel to the beam central axis. The phantom was abutted with solid water to provide full scatter equilibrium. Films were irradiated for both 6 and 9 MeV electron beams delivered with a Varian iX linear accelerator (Varian Medical Systems, Palo Alto, CA) using a 10 × 10 cm cone at 100 cm source‐to‐surface distance.

Film dosimetry was performed adhering to recommendations in AAPM TG‐235.^14^ The films were scanned in transmission mode 24 h post irradiation using an Epson Expression 10000XL scanner (Epson, Los Alamitos, CA). PDD curves were obtained by extracting pixel value profiles of the scanned films using ImageJ software.^15^ A triple‐channel dosimetry method was used to convert pixel values to dose using calibration curves previously established for the film lot. The uncertainty in the film measured dose was calculated to be 3.1% by adding in quadrature recommended measurement uncertainties provided in TG‐235.

The phantom was scanned using a GE Discovery CT Simulator (GE Healthcare, Chicago, IL) with a 1.25 mm slice thickness. The scan was used to replicate the irradiation setup in the Varian Eclipse v15.6 TPS, shown in Figure 1. Dose was calculated using the Electron Monte Carlo (EMC) algorithm for both the native HU of the scanned phantom, as well as manually overriding the phantom with 300 HU, corresponding to the measured physical density of the phantom of 1.18 g/cm^3^. Central axis dose profiles were extracted to compare to the film measured PDDs, and the depths for R90 (depth of 90% of the dose maximum), R80 and R50 were compared.

### 2.4 Clinical case study

The presented 3D printing solution was used to create a bolus for a patient undergoing treatment for basal cell carcinoma of the nose. A CT scan of the patient was acquired and imported into the Eclipse TPS to create a virtual 0.5 cm thick bolus covering the entire nose. The DICOM structure set was export to Adaptiiv 3D bolus software (Adaptiiv Medical Technologies, Halifax, NS, Canada) to create an .stl file of the bolus, which was then printed using the process detailed in Section 2.2. The patient was brought back in for CT simulation where a planning scan was acquired with the bolus fitted on the patient. A VMAT plan using 6X was created in Eclipse to treat the PTV to 5500 cGy in 22 fractions.

## 3 RESULTS

**FIGURE 2:**
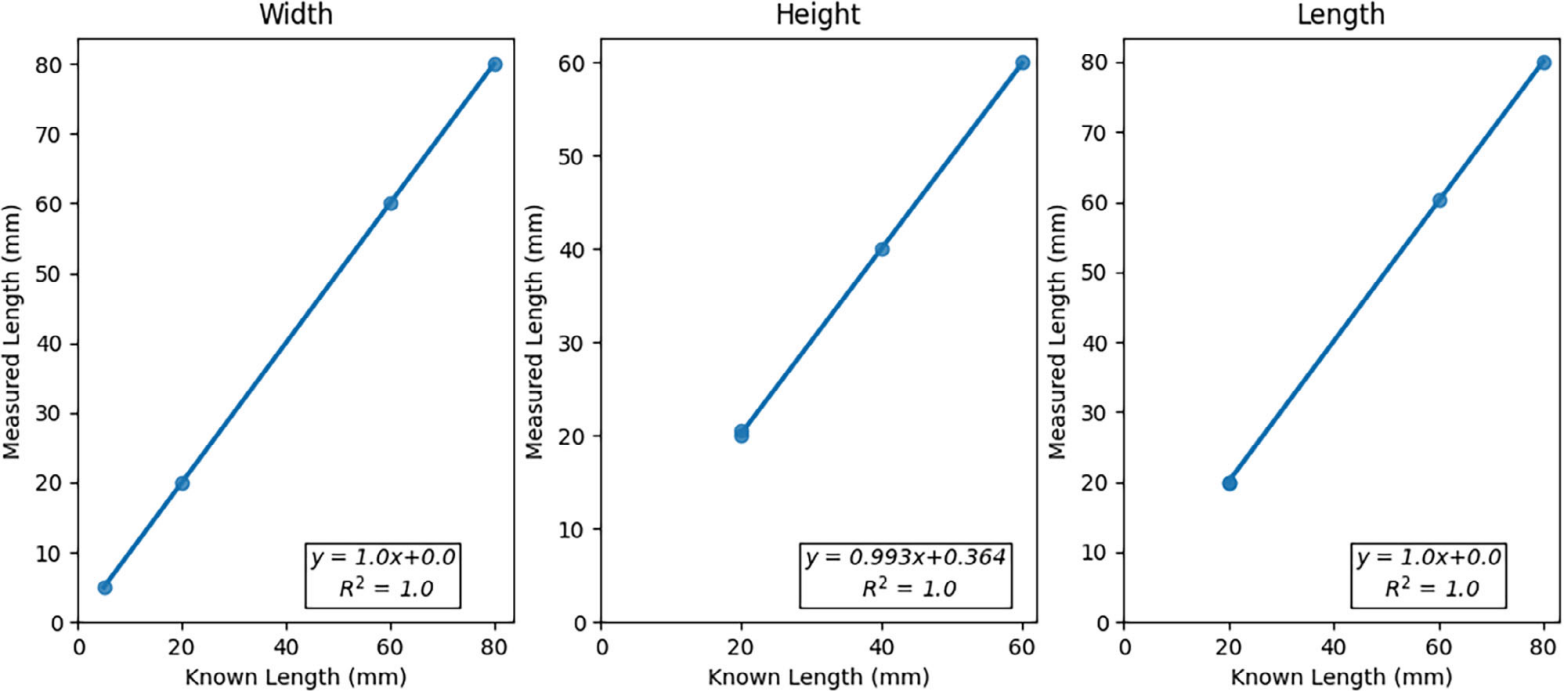
Plots of measured versus known dimensions for the width, height, and length of the test cuboids with corresponding line of best fit.

Plots of the planned dimensions versus the measured printed dimensions for the width, height and length of the test cuboids are presented in Figure 2. Overall dimensional accuracy of the 3D‐printer was very good, with all measured dimensions within 0.5 mm of nominal. The scaling linearity was also good, as demonstrated by the R^2^ value of 1.0 for all dimensions.

The average density of the test cuboids was calculated to be 1.18 g/cc, which was defined as the nominal density of the Premium Flex material.

**FIGURE 3:**
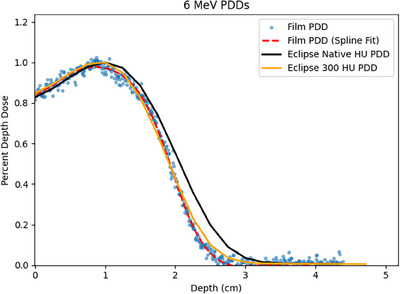
Plot of film measured, native TPS, and 300 HU overridden PDDs for 6 MeV.

**FIGURE 4:**
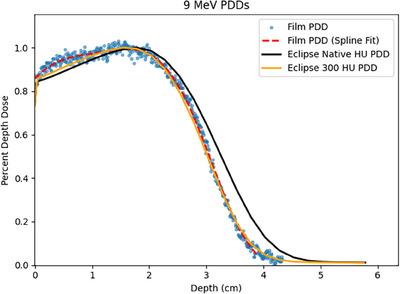
Plot of film measured, native TPS, and 300 HU overridden PDDs for 9 MeV.

Measured versus Eclipse TPS calculated PDDs are presented in Figures 3 and 4 for 6 and 9 MeV, respectively. The average HU measured in the CT scan of the phantom blocks was 105 HU, corresponding to a density of 1.09 g/cc. This density is less than the measured density of the premium flex material, which is illustrated by the poor agreement of the native HU Eclipse TPS PDD curves versus the film measured PDDs. When the HU of the phantom was overridden to 300 HU to correspond to the measured density of 1.18 g/cc, comparisons of the Eclipse TPS PDD versus the film measured PDD showed excellent agreement. Table 2 presents comparisons of R90, R80, and R50 for the film measured PDDs and 300 HU override Eclipse TPS PDDs. The agreement was very good, with all calculated depths within 1 mm of the film measured depths.

**TABLE 2:** Comparison of film versus calculated R90, R80, and R50 for 6 and 9 MeV.

	6 MeV			9 MeV		
	Film measured (cm)	Eclipse 300 HU override (cm)	Difference (cm)	Film measured (cm)	Eclipse 300 HU override (cm)	Difference (cm)
R90	1.16	1.26	0.1	2.19	2.28	0.09
R80	1.49	1.53	0.04	2.54	2.51	−0.03
R50	1.92	1.91	−0.01	3.04	3.02	−0.02

**FIGURE 5:**
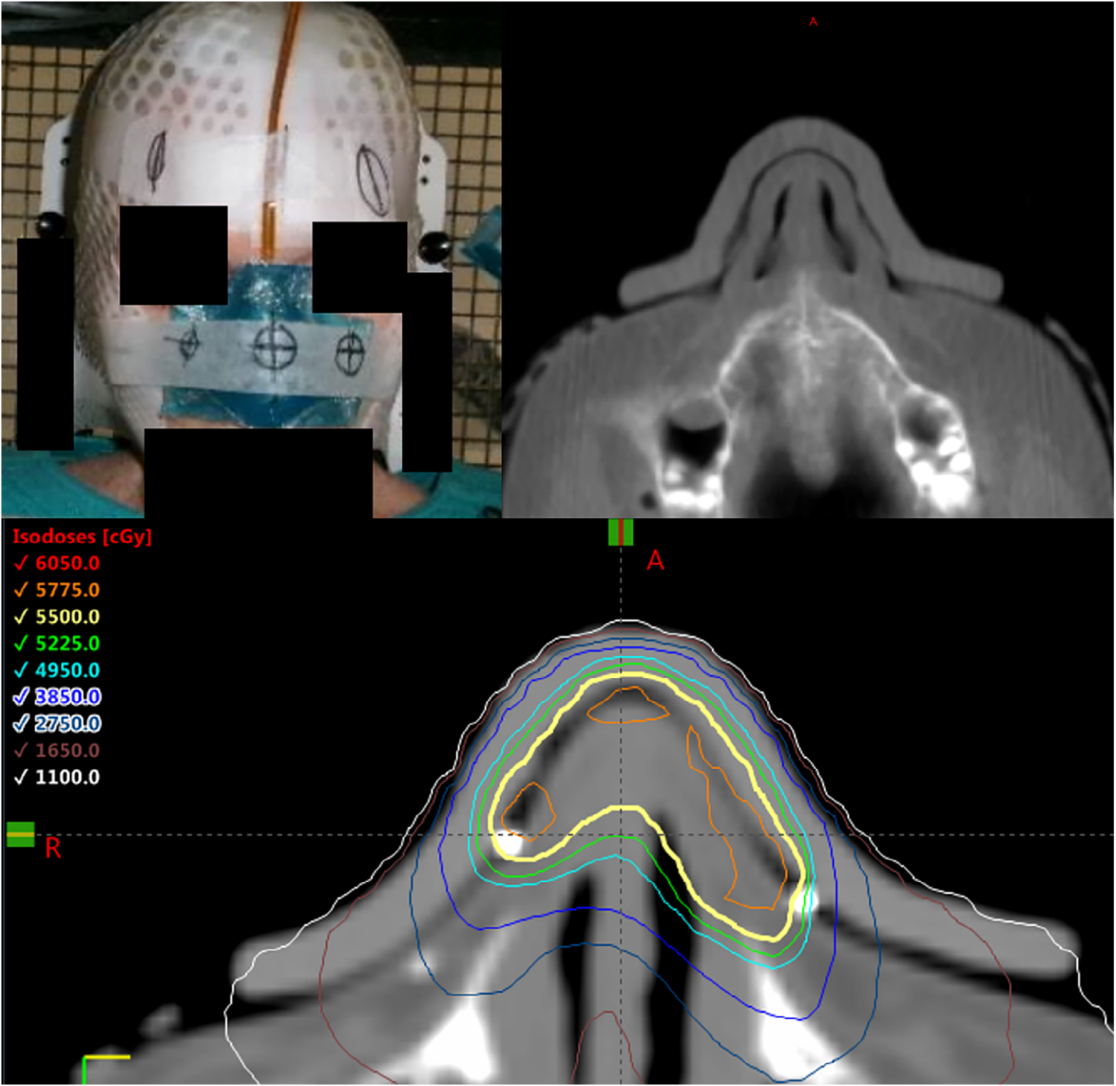
Photos of the clinical case study. Top left image shows the 3D printed bolus fitted on the patient. Top right image shows the planning CT of the patient with the bolus fitted. Bottom image shows the isodose distribution for the VMAT plan.

Figure 5 shows images of the clinical case study. The bolus was 38.7 cc and took 5.5 h to print. The density of the printed bolus was 1.19 g/cc, which was within expected tolerance of the nominal density. Visual Inspection of the bolus on the planning CT demonstrated it to be uniform and free from any inhomogeneities. The bolus was comfortable for the patient and was highly conformal to the patient's anatomy as demonstrated in the planning CT scan in Figure 5.

## 4 DISCUSSION AND CONCLUSIONS

One of the primary benefits of the solution presented in this work is the much lower cost for directly 3D‐printing flexible bolus compared to previously presented solutions. PolyJet 3D‐printers range in price from $30 000 to $500 000+, making them prohibitive for most community clinics to purchase and use in‐house. The LCD SLA 3D‐printer used in the current work was obtained for $1000 and has a print volume large enough to print bolus for most clinical use cases outside of large post mastectomy chest wall bolus. For clinics that only envision needing to print smaller boluses on the order of 15 cm^3^, smaller sized printers can be obtained for as low as $300. The material used in the presented solution is also affordable, at an approximate cost of $150 per 1L.

Total fabrication time and effort is a major concern in the clinical environment. Mold based solutions require 3−6 h to print the mold, 40 min to prepare the mold and silicone, and after pouring the silicone at least 4 h for the silicone to cure, and finally any additional time for removing the bolus and post‐processing.^7^ The LCD SLA 3D‐printer solution presented requires 3−7 h to print the bolus depending on its size, and approximately 40 min for post‐processing. This is about 40% less time compared to mold‐based solutions and involves substantially less manual intervention and labor.

One important consideration of the presented solution is the possible toxicity to the skin of the 3D‐printed bolus. The liquid form of most 3D printing resins is considered toxic for both ingestion and causing skin irritation. After a resin 3D printed part has been fully UV cured, it is generally considered safe to handle. However, the specific material used in the current study has not been ISO tested for prolonged skin exposure. Therefore, it is recommended to line the patient facing side with a layer of plastic cling wrap to prevent direct contact of the bolus with skin.

In conclusion, we have developed and commissioned a low‐cost solution for directly 3D printing flexible bolus in‐house. The SLA 3D printer demonstrated excellent dimensional accuracy and scaling linearity. The measured PDDs of the flexible resin material were in excellent agreement with the Eclipse TPS calculated PDDs when the HU of the material was overridden to 300 HU, which corresponds to the physical measured density of the material of 1.18 g/cc. A clinical case study demonstrated highly conformal bolus can be printed using this solution. Based on these results, the solution can be used clinically for directly 3D printed flexible bolus.

## AUTHOR CONTRIBUTIONS

Garrett C. Baltz is responsible for the study design, acquisition and analysis of the data, and writing of the manuscript. Steven M. Kirsner is responsible for study design, analysis of data, and editing of the manuscript.

## CONFLICT OF INTEREST STATEMENT

The authors declare no conflicts of interest.
